# Demographic and generational determinants of Poles’ participation in the sharing economy: Findings from a survey data analysis

**DOI:** 10.1371/journal.pone.0265341

**Published:** 2022-06-09

**Authors:** Izabela Warwas, Aldona Podgórniak-Krzykacz, Justyna Wiktorowicz, Joanna Górniak

**Affiliations:** 1 Department of Labour and Social Policy, Faculty of Economics and Sociology, University of Lodz, Lodz, Poland; 2 Department of Economic and Social Statistics, Faculty of Economics and Sociology, University of Lodz, Lodz, Poland; 3 Department of Logistics and Informatics, Faculty of Economics and Sociology, University of Lodz, Lodz, Poland; University of Pisa, ITALY

## Abstract

The fast development of technologies in today’s world is accompanied by the mushrooming of digital platforms constituting the core of the ecosystem of sharing economy. This multifaceted phenomenon and its ever-increasing presence have become a subject of public interest and debate, as well as encouraging research and scientific discourse. The article presents the results of the first study of Poles’ participation in sharing economy derived from a questionnaire survey of a representative sample (n = 1000). The purpose of the study was to characterise Poles participating in the digital economy and to determine how they differ in the use of sharing platforms depending on their age group and generation. The analysis has shown that the rates of Poles participating in the digital economy are the smaller, the older the age group, and that a rising number of the users of digital economy solutions translates into greater acceptance of sharing platforms. Among the oldest Poles, 70% do not participate in the digital economy and as much as 80% in the sharing economy. The numbers sharply contrast with generations Z and Y that participate in the sharing economy almost without exception. The most popular of sharing services turned out to be accommodation reservation indicated by every third respondent.

## Introduction

There are two main engines that drive the global economic development: globalization and technology. The pace and complexity of technological progress observed in recent years have an unprecedented scale. Silicon chips, desktops, the Internet or mobile technologies are now giving way to learning technologies and artificial intelligence. Increasing data transmission rates and sophistication of algorithms allow integrated IT architectures to learn, and processes become increasingly adaptive, data-driven and “self-tuning” [1, 2].

The technologies have enabled the emergence and development of a variety of digital platforms making part of the ecosystem of the sharing economy (SE), which allow their users to acquire or offer a short-term use of goods or services. Thus, internet-facilitated platforms make it possible for people to share their underutilized assets [3] and help materialise the idea of consumption based on sharing. They are the emanation of a socio-cultural phenomenon of sharing assets with others, which emerged with the spreading conviction that being able to use things when they are needed is better and more economic than actually possessing them [4, 5]. The SE is a fast-growing and heavily debated phenomenon [3] all over the world, including Poland.

Because sharing platforms belong to the virtual world and digital economy (DE) [6, 7] and include peer-to-peer or business-to-consumer digital transactions, digital competencies and skills and some experience of online activity are necessary to use them. Studies show that older people differ from younger ones not only in the frequency but also in the manner of using information and communication technologies (ICT) [8]. A digital gap between the oldest (65+) and youngest users of the Internet is indisputable. It is mainly attributable to the lower digital skills of older persons [9], which additionally grow obsolete with the steady advancement of digital technologies [10]. Their misconceptions about the Internet and ICT [11] and technophobia discourage them to some extent from using these technologies and determining the patterns and complexity of their use [12]. Older adults in Europe use the Internet much less often than younger people, but even among them, 84% do this more than once a day (especially persons aged 55–65 years) [13]. The proportion of middle-aged users of the virtual world is steadily increasing, likewise of technologically-advanced older people [14].

The digital participation of older people is carefully studied today because of the indisputable benefits it can offer them. It can improve the quality of their lives [15], make them feel less lonely [16, 17], increase their social interactions and social capital [18], the sense of belonging [19], as well as make them more active and independent [20, 21]. The use of computers and the Internet by older people has also been proven to improve their cognitive abilities [22]. The first studies of SE participation of the oldest adults (>85 years) indicate that it can profoundly improve their and their carers’ situation [23].

This article considers how age as a demographic factor relates to the popularity of digital platforms among the different generations of their users. The research process was aimed to answer the following questions:

Q1. Do age and generations (BB, X, Y, Z) have a significant effect on Poles’ decisions to use sharing platforms as consumers?
Q2. Are age and generation (BB, X, Y, or Z) related to preferences of Poles’ choices which sharing platforms they will use as consumers?
Q3. Is gender related to the decisions of particular generations of Poles on whether and which sharing platforms to use?
Q4. Does participation in the digital economy (use of e-banking and e-commerce services) have a significant effect on the decisions to use sharing platforms?

The article has the following structure. The next section contains an analysis of the literature aimed to identify the motivations, attitudes and characteristics of the users of the SE and to find out how their age influences the frequency and motivations for using digital platforms. In Section Materials and Methods, the research method and the survey data are explained. Section Results presents the results of the research into the participation of different age groups of Poles in the DE and the SE and the relationships between them. Lastly, the characteristics and preferences of the Baby Boomers (BB), X, Y and Z generations important for the use of digital and sharing platforms are considered.

## Literature review

**Fig 1:**
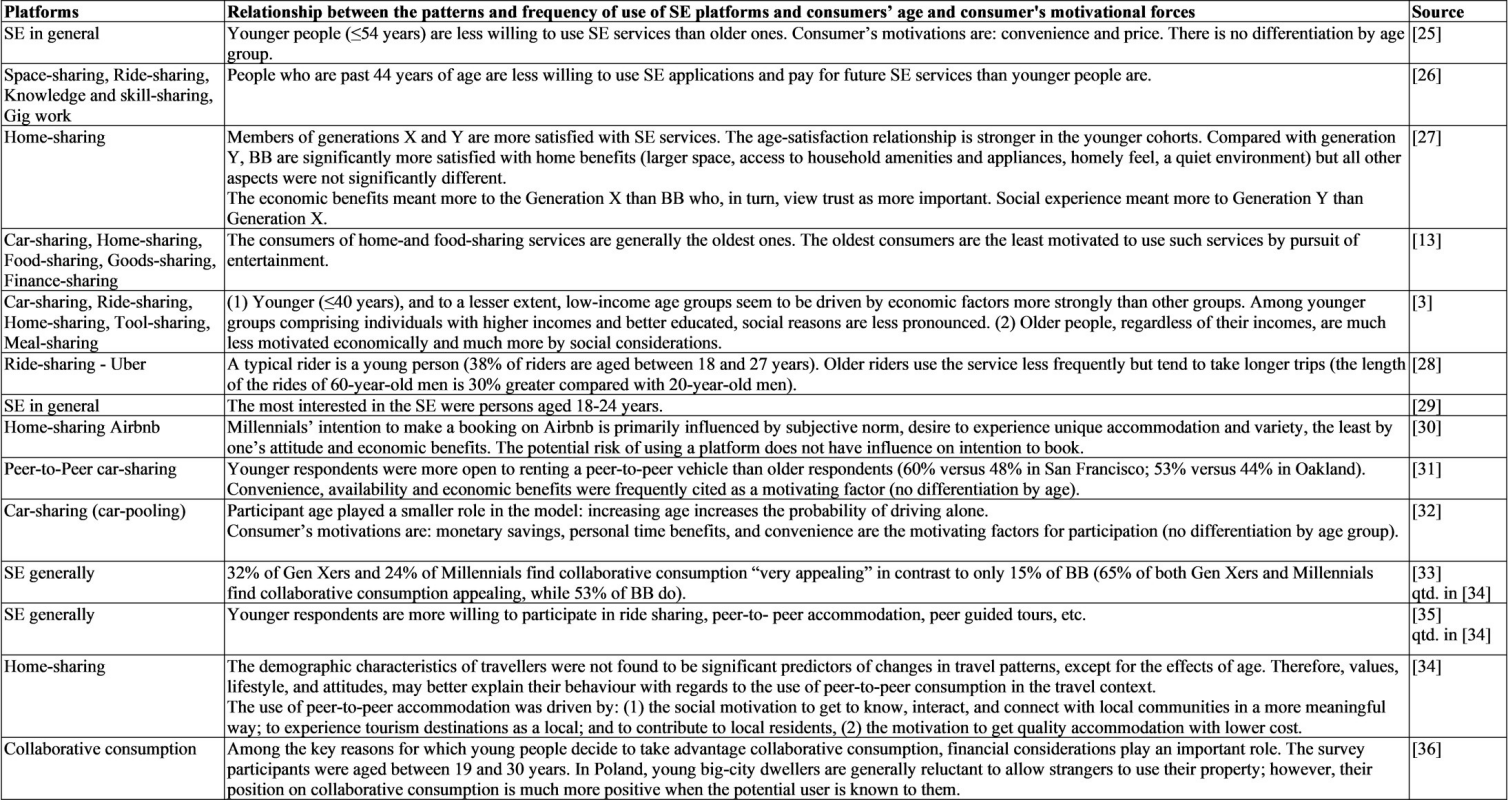
Age and generational membership as factors in the use of SE platforms–an overview of research results [3, 13, 25–36].

The review of previous SE studies (Fig 1) shows that few of them considered whether and how the age group and generation of the potential users of sharing platforms related to their actual use.

According to studies conducted mainly in the US and European markets (primarily in EU western countries, eg. Germany, Scandinavian countries), SE users are age-diverse and come from different generations. Although their results are not conclusive for age groups younger than 45 years (Fig 1), they leave no doubt that the SE participants come from younger cohorts. In this respect, they support the conclusion from preliminary research derived by Pawlicz [24] from an analysis of scientific articles that SE participants are younger than the rest of the population and that they are mostly male. Fig 1 also shows that the users of home-sharing and food-sharing services are the oldest.

In Poland, too, few studies have been conducted to determine how individuals’ age influences their participation in the SE. Based on the assumption, that consumer behavior can be determined by internal and external factors, in the structure of internal behaviors, personal and demographic were distinguished, e. g. age or gender. Concerning age, there was a tendency to decline in the willingness to use tourist services in the context of the SE with the age of the respondents [37]. Chudzian [38] has reported that the rate of active SE users is by far the lowest in the age group 18–25 years and increases with age (however, he did not study age groups older than 40 years). Chudzian’s study is rather exceptional, given that Polish researchers tend to focus on theoretical analyses, reviews, and conceptual analyses [39–44]. The research review shows that there is a lack of detailed analysis regarding the participation of different generations in different types of SE platforms. Our research fills this gap.

Given that studies point to an association between age and the use of sharing platforms, a question arises about how the characteristics of different generations influence their inclination to use the SE platforms. The Baby Boomers (BB) generation appreciates values such as independence, commitment and global thinking, and its main strengths include patience, responsibility, communication skills, the ability to cope with difficult situations, willingness to sacrifice, and great knowledge and experience of life [45]. Having witnessed technological changes during their lifetime, people of the BB generation can be efficient users of modern technologies [46].

Generation X is technologically savvy and is credited with moving the Internet into the mainstream. Its members generally tend to value personal development, independence, diversity, initiative, diligence, flexibility, and entrepreneurship. Their strengths include adaptability, techno-literacy, independence, creativity, global thinking, pragmatism and balance [47]. Generation X seems to be motivated intrinsically rather than extrinsically [48] and can exceed expectations and deliver results [17]. Generation X volunteers join local organizations in greater numbers than the BB did in their youth [49].

Millennials (i.e. Generation Y and Generation Z) are people who were brought up in "better times." Their childhood and youth coincide with globalization and universal, everyday access to the Internet. They instinctively turn first to the Internet to communicate, understand, learn, and find, and they constantly update online content [50, 51]. They also frequently use mobile services, treating them as a medium of self-expression [46]. The changes in the field of operating on the Internet are so large within Generation Y that researchers divided into subgroups of pre-social media bloggers from post-social media bloggers [52]. Millennials’ use of digital media and social media makes them potential users of the SE [9]. As younger Internet users, they are significantly more likely to have liberal attitudes aligned with the Internet’s cultural values [53]. Millennials show somewhat divergent consumption patterns when compared to older generations [54]. In choosing new brands they frequently rely on peer recommendations, transmitted directly or through social networking channels [55].

From the foregoing descriptions of generations, it follows that Millennials are the most likely to become the users of the SE. This conclusion is supported by PWC experts, who leave no doubt that Millennials, the digital generation which reached adulthood alongside the fast-spreading use of modern technologies and increasing availability of the Internet, is the force driving the SE [4]. The SE visionaries also associate its success with the growing up of a generation accustomed to sharing on the Internet, for which sharing is second nature [56].

In Europe, the majority of SE users are young, well-educated people with high digital skills [26, 57–59]. In addition, generations X and Y in Europe present a higher level of adaptation for social networks and e-services [60], which often complement the use of SE and DE platforms. Although aware of the existence of the SE, older Europeans (>45 years) do not engage in it, mainly due to their lower digital competencies [13]. European BB is still behind in terms of Internet access, adaptation for e-services and social networks, digital know-how, and enthusiasm [60]. Studies confirm, however, that the oldest people are also interested in the SE. The Pew Research Centre estimated that in 2016 44% of Americans aged 65 and above used at least one type of an SE platform or on-demand services [61]. The rate is likely to rise following the increase in mobile device and smartphone users in this age group [62].

Studies examining the causes of participation in the SE utilise theories created to explain individuals’ behaviour, among which the theory of planned behaviour (TPB) created by Ajzen [63] is frequently indicated [64–66]. Its core construct is behavioural intention [67] reflecting an individual’s readiness to participate in some activity. The probability of an individual engaging in some activity is the higher, the stronger the behavioural intention. Ajzen’s TPB holds that there are three predictors of behavioural intention: attitude toward behaviour, subjective norm, and perceived behavioural control [63]. The existence of the three predictors has been confirmed by the results of SE studies. Participation in the peer-to-peer platforms that allow access, temporary and paid or free, to the underutilised physical assets or intangible resources of other people mainly depends on individuals’ attitudes and readiness to accept this mode of service delivery [68–70]. Liao et al. [65] argue that the attitude toward the behaviour, subjective norm and perceived behaviour control are the general variables impacting the behaviour intention of SE participants. Kim et al. [64] confirmed a positive effect of the awareness of the SE on attitude toward using sharing services, and this attitude significantly influences consumers’ intention to use sharing services and plays a mediating role in the relationship between awareness of the SE and behavioural intentions. The research argues that the attitude toward the behaviour, subjective norm and perceived behaviour control are the general variables impacting the user’s behaviour.

Factors strengthening people’s behavioural intentions to participate in the SE include a commitment to sustainable development, participation in a social network, and a hope of making new acquaintances. On the other hand, they can be discouraged by the perceived risk of becoming a platform user [70]. The authors of some recent studies point to confidence or a lack of it (especially in the sharing platforms) as a factor important for people’s intention to participate in the SE [26, 68, 69].

The reasons for using a sharing platform also depend on its type (for-profit, not-for-profit) and the product or service it offers, as well as on whether an individual wants to be a customer or a provider. As for consumers, they are mainly guided by financial [13] and practical considerations [71], as well as by a search for fun [72]. According to many studies, financial considerations are the most important [24], especially for people considering the use of for-profit platforms [73]. The financial cost of the service has been found to be more important for the users of the accommodation-sharing platforms than the car-sharing platforms [74, 75]. The former seek a true experience of local life [34] and the latter are motivated by environmental issues [3] or seek ways to cut travelling time [32]. The reasons for using meal-sharing platforms are mainly of social nature [3].

The existence of a relationship between the level of income and participation in the SE has not yet been conclusively confirmed. Some studies have found part of SE participants to be pretty well-off [32, 38, 76], but others report that their incomes are below the median income in their country [34, 72] or have failed to establish an association between the level of income and actual or intended participation in the SE [26]. According to many studies, people with higher education and big-city dwellers in cities are definitely more inclined to participate in SE [25].

There is also a link between age and the motivations of SE users: younger age groups (<40 years), as well as the Generation X compared to BB appear to be more strongly motivated by financial considerations [3, 13, 27]. Comparing Generation X and BB Mahadevan [27] argues, that economic benefits meant more to the former as they may have more financial burden than BB because they are more likely to have financially dependent children along with a parent aged over 65. There is no clear evidence that social considerations motivate older people more than younger ones [13, 27, 30].

This study was designed to learn more about the characteristic of Poles participating in the SE, with a special focus on identifying differences between the generations and age groups of Polish adults. After a review of the literature, a research hypothesis was formulated that Poles’ demographic characteristics such as age and represented generation influence their participation in the SE as well as the selection of sharing platforms. More specifically, it was assumed that people older than 45 years (i.e., Generations X and BB) would be less willing to use the SE platforms than younger ones (Generations Y and Z) and that they would show a preference for the accommodation-sharing platforms, which are the most popular in Poland. As for the younger generations (Y and Z), the assumption was that they would use a large number of different platforms and that car-sharing would be more popular with them, as they travel more often for reasons such as education, work, and social activities.

## Materials and methods

The empirical study was carried out in 2020 in Poland using the CATI technique (computer-assisted telephone interviewing). The study covers the representative sample n = 1000 adult (18 years and over) residents of Poland (with the estimation error of 3%). The questionnaire was short, dedicated to the aim of this study (S1 Questionnaire). It includes first of all questions related to sharing and digital economy, as well as the most important (in the aim of paper context) demographic features (collected data are in the S1 Dataset file).

Regarding the aim of this paper, age and generational differentiation in the digital and sharing economy was analysed. Generational theorists argue that adopting a generational approach yields richer information than one using chronological age and life stage because generational cohort analysis can acknowledge the subjective historical influences of time on human behaviour [77]. In this paper generations are defined as following [78]: BB (baby boomers)–people born between 1946 and 1964 (in 2019, when the methodology of this study was prepared, aged 55–73 years old), X–born between 1965 and 1979 (aged 40–54), Y–born between 1980 and 1994 (aged 25–39) and Z–born in 1995 or later (aged below 25). The sample’s structure from the perspective of these two criteria is presented in Table 1. Regarding different response rates in subpopulations divided by age and sex, analytical wages were used).

**Table 1:** Sample characteristics.

Specification	Total		Number by gender	
	n	%	Women	Men
Total	1000	100.0	521	479
Age (years)				
18–24	110	11.0	54	56
25–34	205	20.5	100	105
35–44	180	18.0	88	92
45–54	151	15.1	77	74
55–64	176	17.6	92	84
65+	178	17.8	110	68
Generation				
Z	354	35.4	202	152
Y	234	23.4	118	116
X	302	30.2	147	155
BB	110	11.0	54	56

In keeping with previous SE studies [79] and conceptual analyses, we considered the use of six sharing platforms offering the following services: (1) accommodation booking, (2) car sharing, (3) free access to goods/services/knowledge/skills, (4) outdoor equipment sharing and exchange, (5) tours guided by locals, (6) crowdfunding.

The selection of these particular platforms was also dictated by their availability in Poland. As most sharing services are paid for online, the use by respondents of electronic banking was also examined in the study, as well as their activity on buy-and-sell online platforms (e-commerce), to assess their participation in the DE. Both activities were recognized as the predictors of their use of the SE.

Regarding behavioural intentions concept, the degree of Poles’ openness to sharing platforms was summary measured as the total number of used SE platforms (SEO–Sharing Economy Openness). The possible range of this variable is [0,6], where 0 means the lack of SE platforms using, and 6 –using all analysed platforms. Kaiser-Meyer-Olkin measure [80] (KMO = 0.701) confirms the adequacy of this variables set. Exploratory factor analysis with principal component extraction method and Kaiser criterium [81] confirms homogeneity of SEO indicator.

Statistical analysis of SEO, their components and digital economy descriptive statistics (M–mean, Me–median, MT–trimmed mean, SD–standard deviation, S–skewness), as well as the chi-squared test of independence, Mann-Whitney test, and Kruskal-Wallis test were used. The listed tests were applied to compare populations according to their electronic platform usage–for each platform separately and for summary assessment of SE openness. For each platform effect size of gender, age and generations was evaluated with V-Cramer coefficient (V). Based on Cohen approach [82], value 0.5 and more can be interpreted as high effect size, between 0.3 and 0.5 –effect size is moderate, between 0.1 and 0.3 –small. Finally, logistic regression was used what allows to estimate the probability of SE openness and its determinants. The logistic regression equation was estimated using the maximum likelihood estimation method. The logistic regression models were considered correct if: (1) in the omnibus test of model coefficients p < α, (2) in Hosmer-Lemeshow test p > α, (3) Nagerkelke’s pseudo R2 is relatively high, (4) quality of classification is relatively high, in particular, the percentage of correct qualifications for y = 1 and count R2 are high [83]. For all calculations, we adopt a standard level of significance (α = 0.05).

## Results

### Poles’ participation in the DE

The distribution of adult Poles’ answers to the question about the use of online services shows that almost one-third of them do not use them (Table 2). The most popular online services are the sale and purchase of goods used by almost two-thirds of the adult population in Poland (63.1%) and electronic banking used by an insignificantly smaller proportion of Poles (63.8%). Both DE services are statistically significantly more often used by men than women and the difference of participation reach ca. 16 pp for electronic banking and ca. 12 pp for online shopping (Table 2).

**Table 2:** Poles’ participation in the digital economy by generation and gender.

Specification	Electronic banking			Purchase / sale of goods		
	Total	Gender (V = 0.174**)		Total	Gender (V = 0.120**)	
		Female	Men		Female	Men
Total	63.1	55.1	71.9	63.8	58.2	69.8
Generations						
Z	92.3	95.6	89.4	92.3	95.5	89.4
Y	90.4	84.9	95.1	91.1	87.0	95.8
X	69.2	61.0	77.4	75.3	68.3	82.3
BB	31.9	24.0	42.4	29.4	26.9	32.7
V	0.535**	0.563**	0.496**	0.576**	0.562**	0.598**

** p < 0.01.

The generational differences are also statistically significant and its (generation’s) effect is strong (V-Cramer coefficient equals 0.5–0.6). The distribution of answers (Table 2) shows that almost all generations Z and Y participate in the DE. In X generation, a lower rate of this participation, ca. 70%, is observed. The BB generation is the one that is the most cut off from the digital, where the rate of non-users reaches around 70%. There are more women from the Z generation participating in the DE than men; in the other generations, men are more active than women. In BB generation online shopping is similarly popular for men and women (Table 2). The rates of Poles participating in the DE decrease with their age (Table 3).

**Table 3:** Poles’ participation in the digital economy by age groups.

Platforms	18–24	25–34	35–44	45–54	55–64	65+	V
Electronic banking	92.3	92.7	80.2	66.7	42.3	23.8	0.546**
Purchase/sale of goods	92.3	92.2	85.3	71.7	40.5	21.0	0.589**

** p < 0.01.

In the under-35 age group, only a few percent of Poles (7–8%) do not use online services, after 45 percent of DE users is significantly lower (Table 3). It’s worth noting that the highest participation in electronic banking takes place in the 25–29 group (98.8%), for people aged 55–59 decreases to 50.6%, and for 60–64 group–to 35.2%. Similar tendencies are observed in the case of online shopping (percentages for the above groups are: 95.1%, 45.0%, and 36.0%). Summarising, participation in DE is the highest for young people, especially aged 25–29 years old, and decreases with age, with a higher decrease after 50. In the non-productive age, the interest in DE tools is low, which is typical for many countries and is related to lower ICT skills [84].

### Using of sharing platforms in Poland

**Fig 2:**
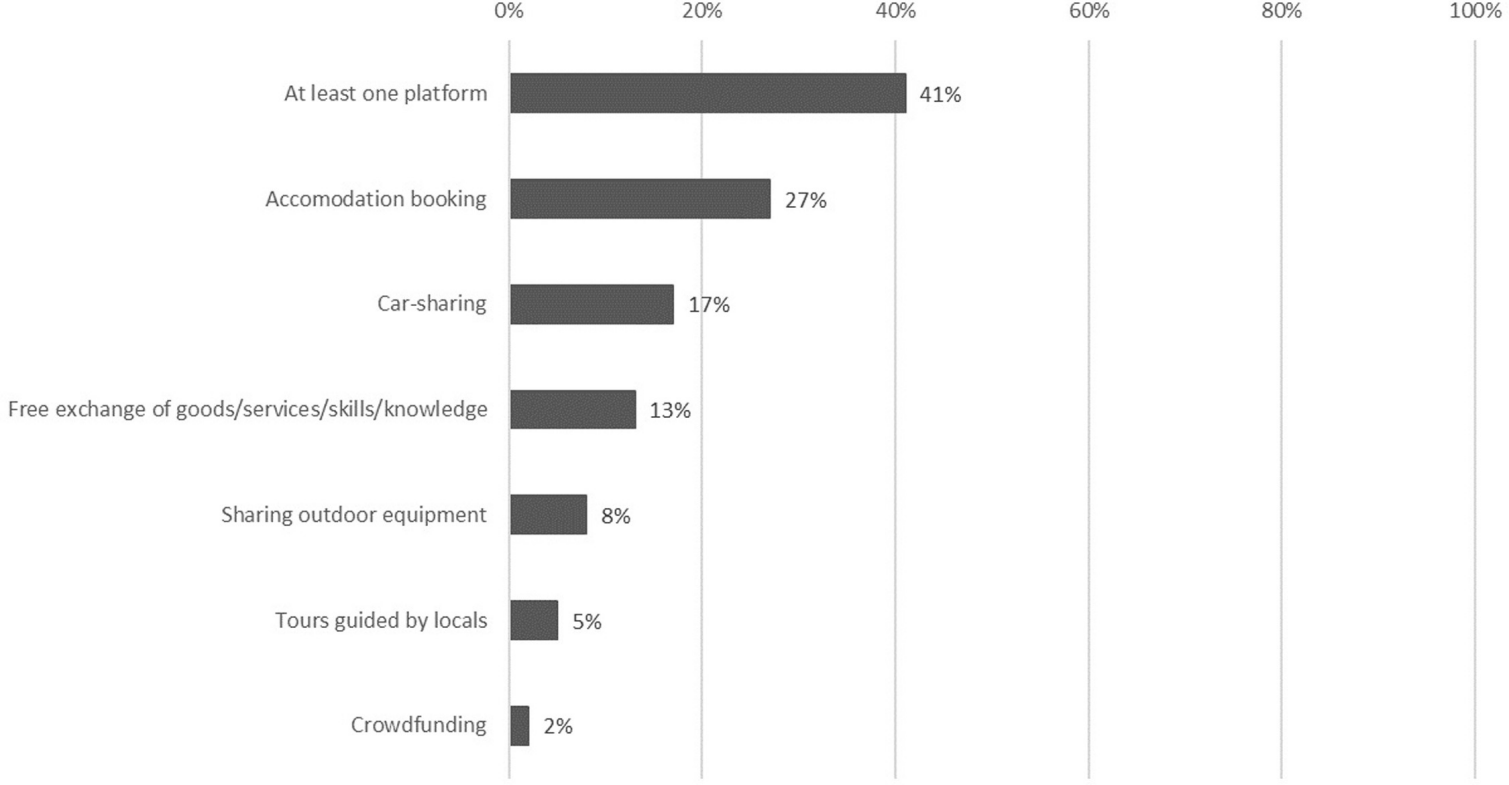
The ranking of sharing platforms by frequency of use (n = 1000, %).

The popularity of sharing platforms among Poles is varied for different services (Fig 2). At least one type of sharing platform is used by ca. 40% of Poles. The most popular are platforms offering accommodation booking services, which usage was declared by 26.7% of respondents. Car-sharing and the free exchange of goods/services/knowledge/skills ranked in the middle, having been indicated by 17.0% and 13.0% respondents, respectively. Sharing platforms allowing their users to borrow or lend swap outdoor equipment appear not to be very popular in Poland (7.7%). Let us note that not all cases when such equipment is lent or exchanged involve sharing. Tours guided by locals were declared by 5.5% of respondents, and merely 2.3% availed themselves of crowdfunding platforms to raise funds for charitable projects, start a business, buy a gift for oneself or another person, or finance one’s dream trip. Thus, the “pure” sharing services are less popular among Poles.

The usage of the SE platforms is related to age and this conclusion applies to SE in general as well as to each service. This relation is statistically significant and the size effect is moderate for SE in total as well as for accommodation booking and car-sharing (for other SE services this relation is lower, but still significant). The participation in at least service declared 2/3 of young people (before 35) and 40–50% aged 35–44, towards only ¼ people aged 55–64 and 13% aged 65+ (Table 4). Similar conclusions (lower and lower percentage with age and small differences between two young groups) are suitable also for each service, excluding crowdfunding. Crowdfunding is the most specific for people aged 35–44, it’s often used also in the education period (before 25). The most popular SE services are (in respect of age): accommodation booking, used especially often by Poles aged 25–34 (44%) and 18–24 (39%). For the last group, similar popularity has car-sharing (40%). Whereas, free exchange of goods, etc., are preferable by Poles aged 35–44 (20%). Generally, the participation in SE platforms significantly decreases for the population aged 65+ and this conclusion applies also to accommodation booking, free exchange of goods/services/skills/knowledge, and crowdfunding. Age 60+ is such a “cut-off point” in the case of car-sharing, and 55+—for sharing outdoor equipment and tour guided by locals.

**Table 4:** Poles’ participation in the sharing platform services by age groups (%).

Platforms	18–24	25–34	35–44	45–54	55–64	65+	V
At least one platform	63.7	64.8	49.5	40.8	26.9	13.1	0.388**
Accommodation booking	39.1	43.8	31.5	28.3	18.5	7.9	0.284**
Car-sharing	39.6	27.0	22.4	14.5	8.3	2.3	0.304**
Free exchange of goods/services/skills/knowledge	16.5	16.9	19.8	15.8	8.3	3.3	0.184**
Sharing outdoor equipment	13.2	12.8	11.7	8.6	1.8	0.9	0.194**
Tours guided by locals	11.0	10.1	6.1	5.9	1.2	1.9	0.155**
Crowdfunding	3.3	1.7	5.6	1.3	1.8	0.5	0.119*

** p < 0.01;

* p < 0.05.

Statistically significant are also differences between women and men (Table 5). At least one SE tool is used by half of the men and one in three women. The highest differences refer to sharing outdoor equipment and crowdfunding (for women the percentages are approx. Three three times higher than for men), but also for other SE services percentage of users is 1.5–2 times higher for men.

**Table 5:** Sharing platforms’ services used in Poland by generation and gender (%).

Specification	At least one SE	Free exchange of goods/ services/skills/ knowledge	Crowdfunding	Car-sharing	Accommodation booking	Sharing outdoor equipment	Tours guided by locals
By gender								
Women	33.3	9.9	1.3	14.0	20.7	3.6	3.8
Men	48.8	16.3	3.4	20.3	33.3	12.2	7.1
V	0.158**	0.095**	0.067*	0.084**	0.143**	0.160**	0.073*
By generation: total								
Z	63.7	16.5	3.3	40.2	39.6	13.3	11.0
Y	61.8	19.6	3.2	27.4	40.2	12.8	9.3
X	41.5	15.4	2.4	15.8	28.3	8.9	5.3
BB	19.4	5.8	1.0	5.0	12.6	1.3	1.3
V	0.379**	0.159**	0.238	0.308**	0.271**	0.194**	0.162**
By generation: women								
Z	64.4	18.2	2.2	44.4	37.8	9.1	9.1
Y	55.1	13.8	1.7	23.7	34.1	6.5	5.8
X	31.7	12.2	0.8	11.4	21.1	3.3	4.9
BB	13.8	4.6	1.4	3.2	8.3	0.5	0.9
V	0.406**	0.199**	0.910	0.357**	0.288**	0.164**	0.139*
By generation: men								
Z	63.0	14.9	4.3	36.2	41.3	17.4	12.8
Y	68.3	25.4	4.9	31.0	46.2	18.9	12.7
X	51.2	18.7	4.1	20.2	35.5	14.6	5.7
BB	26.7	7.3	0.6	7.3	18.2	2.4	1.8
V	0.349**	0.176**	0.139	0.268**	0.247**	0.218**	0.184**

** p < 0.01;

* p < 0.05.

Statistically significant are also differences between generations–analysed in total and separately for women and men (Table 5). The only exception is crowdfunding–the percentage of users is similar for all generations. The highest effect size is for car-sharing and accommodation booking, especially for women (respectively, V = 0.357 for car-sharing and V = 0.288 for accommodation platforms). At least one service was used by approx. 2/3 Poles from Z and Y generation, but for women higher percentage is in Gen Z (64.4%) and for men–in Gen Y (68.3%). Men are more interested than women in using these services also in X and BB generations, for the last one this participation is two times higher for men (Table 5). The highest percentages can be noted for accommodation booking for men from Y (46.2%) and Z (41.3%) generations, as well as for car-sharing for women from Gen Z (44.4%).

The online accommodation booking platforms are used by similar proportions of Z and Y generations, but they are used by only 13% of the BB (a rate twice higher compared with Gen X) which clearly points to the treading generations.

Generation Z has the highest rate of peer-to-peer car-sharing platform users (40.2%). The Generation Y rate is below 30%, and in the next generation (X) it is lower by half–ca. 15%. The peer-to-peer goods/services/skills/knowledge exchange platforms users belong mainly to three generations–Z, Y, and X (15–20%). Other platforms, through which outdoor equipment can be borrowed or lent or the services of local guides can be obtained, are mainly used by the representatives of Z and Y generations (ca. 13% and ca. 10%, respectively).

Men from Generations Y and X use sharing platforms slightly more often than women. Among the BB, the male users predominate too, except for crowdfunding platforms that are used slightly more often by women. The use of sharing platforms by Generation Z is more complex. While more women use platforms enabling free exchange goods /services/skills/knowledge and peer-to-peer car-sharing services, for all other platforms men predominate.

The above findings corroborate our hypothesis that there is a relation between the age characteristics of Poles (age groups and generational membership) and their use of sharing platforms. People aged 45+ (Gen X and the BB) are less interested in the platforms than those below 44 years of age (Gen Y and Z). Moreover, individuals’ age and generational membership determine which platforms they will use. Our assumptions that the accommodation booking platforms are mainly used by people aged 45+, whereas car-sharing platforms are the most popular among the youngest and very mobile people, have been confirmed as well.

### Poles’ openness to sharing platforms

Regarding behavioural intentions concept, the assessment of the degree of Poles’ openness to sharing platforms using SEO indicator was made (main results in Table 6).

**Table 6:** Openness to sharing platforms.

Specifi-cation	% of users (by number of SE platforms)							Descriptive statistics for SEO					
	0	1	2	3	4	5	6	Me	M	MT	SD	S	p
Total	59.3	20.7	11.6	6.2	1.8	0.2	0.2	0	0.72	0.60	1.06	1.57	n.a.
By age groups													
18–24	36.4	23.6	25.5	9.1	5.5	.	.	1	1.24	1.15	1.20	0.65	<0.001**
25–34	35.2	32.4	19.4	11.0	2.0	.	.	1	1.12	1.06	1.08	0.68	65+ < ALL
35–44	50.4	22.6	14.9	7.2	2.7	1.1	1.1	0	0.97	0.82	1.28	1.54	55–64 < 18–44
													45–54 < 18–34
45–54	59.0	21.0	8.2	9.8	2.1	.	.	0	0.75	0.64	1.09	1.36	
55–64	72.9	17.9	6.2	2.5	0.5	.	.	0	0.40	0.29	0.76	2.14	
65+	86.8	9.7	2.8	0.6	.	.	.	0	0.17	0.09	0.49	3.17	
By gender													
Women	66.8	18.8	9.8	3.7	0.7	0.2		0	0.53	0.42	0.89	1.79	<0.001**
													M > W
Men	51.1	22.7	13.6	9.0	3.0	0.2	0.5	0	0.92	0.81	1.19	1.29	
By generations: total													
Z	36.4	23.6	25.5	9.1	5.5	.	.	1	1.24	1.15	1.20	0.65	<0.001**
Y	38.2	30.0	18.3	9.6	2.7	0.7	0.4	1	1.12	1.02	1.18	1.06	BB < ALL
X	58.5	20.4	10.1	8.8	1.7	.	0.5	0	0.77	0.65	1.12	1.54	X < Z,Y
BB	80.7	13.3	4.3	1.4	0.2	.	.	0	0.27	0.17	0.53	2.67	
By generations: women													
Z	35.2	25.9	25.9	9.3	3.7	.	.	1	1.20	1.13	1.14	0.62	<0.001**
Y	44.9	34.1	14.1	5.4	0.8	0.8	.	1	0.85	0.75	0.99	1.32	BB < ALL
													X < Z,Y
X	68.2	16.3	9.7	5.1	0.7	.	.	0	0.54	0.43	0.92	1.69	
BB	86.4	9.1	3.7	0.8		.	.	0	0.19	0.10	0.53	3.06	
By generations: men													
Z	37.5	21.4	25.0	8.9	7.1	.	.	1	1.27	1.19	1.26	0.67	<0.001**
													BB < ALL
Y	31.7	26.0	22.4	13.7	4.7	0.7	0.7	1	1.39	1.29	1.29	0.78	X < Y
X	48.9	24.4	10.5	12.5	2.7	.	0.9	1	0.99	0.88	1.26	1.31	
BB	73.3	18.9	5.1	2.3	0.5	.	.	0	0.38	0.27	0.73	2.25	

p—probability in Mann-Whitney test (for gender) or Kruskal-Wallis test (for age groups and generations);

** p < 0.01.—‘percentage of users’ in the sample equals 0.

Only a very small percentage of the respondents (only 0.4% of the total population) declared using all or nearly all SE platforms (all of them were 35–44 years old, most of them–men, none of them are representants of Gen’s Z and BB). Approx. 60% of respondents don’t use any SE platform and this share is the highest for people aged 65+ (86.8%), BB generation (80.7%), especially BB’s women (86.4%). Means (M and MT) of the SEO variable are low–for the total population the trimmed mean is below 1 (0.6 with SD = 1.06), and the median equals only 0. The skewness is strong, right-tailed (Table 6). The young people (aged below 35, from Gen Z and Y) often use at least one SE platform and the number of them is the highest–median at the level 1, trimmed mean–over 1 (for women the highest means are for Gen Z, for men–for Gen Y). Both age and generations, as well as gender, differentiate the scale of SE services usage (p < 0,001*). BB have a statistically significant lower number of used SE platform than other generations (in total and for women and men), for women also Gen X has lower results than Z and Y generations, for men–only than Gen Z. By age, people 65+ have lower SEO level than all younger age groups, people aged 55–64 –lower than those aged 18–44, and people aged 45–54 –lower than 18–34. The highest means (M, MT, Me) for SEO variable are in the Gen Z, aged 18–24 (for men in Gen Z), higher results were obtained for men than women.

Analysing SE openness determinants, logistic regression models were built. The output variable was the dichotomous variable SEOD (y = 1 for people using at least one SE platform, 0 –others). In the role of the independent variable using each of DE services (e-banking and e-shopping variable, dichotomous ones). Additionally gender and one of two measures of age–age groups (model 1) or generations (model 2)–were included.

Taking into consideration the p-value, we can note that the most important for SE openness is the propensity to DE usage (p < 0.001). The probability of SE openness is, ceteris paribus, 5.7 times higher for people using e-banking platforms and approx. 3 times higher for Poles using e-shopping platform (in comparison with non-users). Also gender (p = 0.005) and age (p = 0.014) are statistically significant factors of SE openness. For men, this probability is 1.5 times higher than for women, and in comparison with people aged 65+ in each group this probability is 1.6–2.7 times higher (Table 7). The statistical quality of this model is high–the omnibus test of model coefficient, the Hosmer-Lemeshow test, and Nagelkerke R2 confirm the goodness of fit of this model. Additionally, classification features are high– 85% of people using SE services are classified correctly (count R2 is high, too).

**Table 7:** Probability of the SE openness (SEOD) in the context of the age groups–logistic regression results (model 1).

Specification	B	S(B)	OR	Wald test		
				statistic	df	p
Const	-3.132	0.272	0.044	132.287	1	<0.001**
e-banking	1.740	0.233	5.700	55.640	1	<0.001**
e-shopping	0.986	0.232	2.679	18.090	1	<0.001**
Gender^a^	0.435	0.153	1.545	8.065	1	0.005**
Age groups^b^				14.210	5	0.014*
18–24	0.957	0.339	2.604	7.988	1	0.005**
25–34	1.010	0.300	2.745	11.319	1	0.001**
35–44	0.572	0.292	1.772	3.829	1	0.050*
45–54	0.480	0.305	1.615	2.469	1	0.116
55–64	0.477	0.302	1.611	2.491	1	0.115
Omnibus test of model coefficients			χ2 (8) = 326.2, p < 0.001**			
Hosmer-Lemeshow test			χ2 (8) = 11.2, p = 0.189			
Nagelkerke R2			0.376			
Classification quality for y = 1			85.1%			
Count R2			72.9%			

Reference groups:

^a^ female,

^b^ baby boomers (BB) generation; B, regression coefficient; S(B), standard error for regression coefficient; OR, odds ratio; df, degree of freedom; p, probability in: the Wald test/omnibus test of model coefficient/Hosmer-Lemeshow test;

** p < 0.01;

* p < 0.05.

Analogous results are obtained for e-banking and e-shopping services, as well as for gender when generations are taken into consideration (model 2) (Table 8). Generations’ effect is also statistically significant (p = 0,006). In comparison with BB, Z and Y generations have approx. 2 times higher probability of SE openness. The differences between X and BB generations are not statistically significant (p = 0.449).

**Table 8:** Probability of the SE openness (SEOD) in the context of the generations–logistic regression results (model 2).

Specification	B	S(B)	OR	Wald test		
				statistic	df	p
Const	-2.898	0.221	0.055	171.586	1	<0.001**
e-banking	1.754	0.234	5.778	56.376	1	<0.001**
e-shopping	1.011	0.231	2.749	19.112	1	<0.001**
Gender^a^	0.435	0.153	1.545	8.077	1	0.004**
Generations^b^				12.347	3	0.006**
Z	0.686	0.288	1.986	5.693	1	0.017*
Y	0.644	0.219	1.905	8.686	1	0.003**
X	0.167	0.221	1.182	0.574	1	0.449
Omnibus test of model coefficients			χ2 (6) = 324.1, p < 0,001**			
Hosmer-Lemeshow test			χ2 (7) = 12.6, p = 0.083			
Nagelkerke R2			0.373			
Classification quality for y = 1			85.3%			
Count R2			72.3%			

Abbreviations as in Table 8.

Also, model 2 has good quality. Classification features are high– 85% of people using SE services are classified correctly.

In conclusion, SE openness is related to gender, age and generations–is higher for men than women, and decreases with age and generations. Estimation of this openness’ probability allows concluding that, ceteris paribus, important significance have gender, age/generation as well as DE platforms usage.

## Discussion

Since the book published by Botsman and Rogers [85] ‘sharing economy’ has become a popular buzzword in media [86]. The complexity of this phenomenon is increasingly revealed by metaanalysis and studies, such as this one, which was undertaken to advance the understanding of the SE.

The results of our study confirm low Poles’ openness to SE platforms and the existence of a strong relationship between the demographic characteristics of Polish men and women and their openness to SE and the popularity thereof. The low openness to SEs may still be due to Poles’ limited knowledge about them. The younger they are, the more sharing services they use. A confirmation of this relation can be found in researchers who studied consumption [26, 28, 29, 87], intentions to use [25], motivations [3] or openness to the SE [31].

A significant group among the users of sharing platforms in Poland is generation Z members because they are active on social media and ready to welcome new experiences while having limited financial resources, which compels them to explore less expensive solutions [88]. Only accommodation platforms and platforms enabling the free exchange of resources, knowledge, and skills are used by them slightly less often than the other platforms [13]. The lower popularity of accommodation reservation platforms among people aged 18–24 years than among the age group 25–34 years and among Generation Z compared with Generation Y is probably because the younger groups are less inclined to travel to other cities and countries to avoid travelling costs. As regards platforms for resource (skills, knowledge, etc.) sharing, the use of these requires the possession of assets that take time to be accumulated. Generation Z and those under the age of 24 clearly dominate over the other generations in terms of car-sharing use, which is due to their high mobility and search for cheaper ways to travel. The higher rate for women in this group (44%) than for men can be explained by the more frequent use of private cars by men, but also by the higher environmental awareness of women in this age group [89]. This result also indicates a fairly high feeling of safety among women using this type of service.

Studies show that the popularity of sharing platforms largely depends on the level of trust in society, which underlies collaborative business processes, innovations, and the functioning of social networks without which the SE couldn’t expand. In addition, the information available to assist users in decisions focuses mainly on community-generated trust and reputation information [90]. The level of public trust in Poland is relatively low compared with other nations [91]. It is higher in the older age groups [92], which is inconsistent with the lower use of sharing platforms by older people established by our study. Another category of trust that SE studies should address is trust in technology. Its classification into direct trust and recommendation trust proposed by Alzahrani et al. [90] seem to explain well differences in the use of technology between generations observed in our study. Younger consumers develop trust in new technologies faster, based on recommendations from online social networks. Older people in Poland have less direct trust in technology because they are warier of new solutions and are aware that their digital competencies are limited. They are also less likely to acquire recommended trust. The pattern has been confirmed by a survey of 1,000 adult Poles conducted by IBRIS on commission from the PwC [4]. Answering the question On what basis do you assess the reliability of SE platforms, the respondents pointed to opinions of their acquaintances (60%), opinions circulating in social media (23%), the knowledge of the brand (20%), and the number of stars (14%).

As the main channel through which the knowledge and experiences of sharing platforms are distributed and promoted is the Internet, it is quite unsurprising that older adults who are less active on the Internet are also less engaged in the SE. In this respect, they are worlds apart from Generation Z that mostly consists of trying consumers who personally explore market opportunities, seek new ways to meet their needs, and make choices using their own or other people’s experiences [93]. This behaviour corroborates the earlier statement about direct and recommended trust.

There is one more factor that seems to explain differences in the use of SE platforms by different age groups in Poland. According to B. Grabiwoda [94], while Generation Z consumers exploit the benefits of globalisation with a great skill, they are also increasingly aware of its deleterious consequences. The awareness of the damaging effect of mass production and mass consumption on the environment and of corporate malpractices makes them seek ways to make informed and ethically responsible consumer choices [94]. Hence their focus on the SE platforms as a means for sustainable consumption. The versatility of the platforms also meets their need for group membership and individuality [88], as that meet their individual preferences while giving them the feeling of belonging to the global community. A greater environmental awareness of younger Poles than of people aged 60+ [89] formed by the education system and the Internet can also be an important factor making them use the SE platforms.

The results of our study show that participation in the DE contributes to Poles’ openness to sharing platforms. Among the oldest generation of Polish men and women (BB), only ca. 30% use electronic banking and buy and sell online. This implies that the other two-thirds may lack digital competencies, which may be a major obstacle for them to join the ranks of SE users (ca. 80% of the BB do not use any sharing platforms at all). The cultural and structural backwardness of this generation is, therefore, considerable.

At the same time, however, the fact that almost one-third of the BB participates in the digital economy and, therefore, has sufficient digital competencies to use the sharing platforms implies that there must some other reasons why so few actually use them. One of them can be that older adults need much more time to accept new solutions and innovations. The limited use of sharing platforms may be caused by a gap between their expectations and needs and the functionalities and services offered by Polish sharing platforms. Elsewhere, the first SE platforms specifically dedicated to the oldest consumers (Silvernest, Go Go Grandparent, etc.) are established, or platforms such as Uber or Lyft are provided with functionalities addressing their needs.

The main limitation of our study is that it does not consider the financial and professional situation of the respondents. It is potentially important because older adults’ experience of working in a virtual environment may make it much easier for them to become users of the SE platform(s). In our study, we also did not take into account the size of the town of residence. This is particularly important for older age groups and may be an important control variable that we advocate for inclusion in further research.

## Conclusions

Analysing the age structure of sharing platforms users expands the knowledge about their needs, expectations, motivations, and behaviours in the virtual world. Knowing them is important for promoting the use of sharing platforms among different generations, ensuring a better fit between the providers and consumers of peer-to-peer services, and designing the platforms’ functionalities.

The SE can potentially become a solution effectively supporting active aging among society by facilitating the development of the silver economy, workplace age management, or generation management, advancing sustainable and inclusive employment ecosystems. The oldest users of sharing platforms in Poland can benefit in many ways from them, share their experience and knowledge and support socially important projects, e.g. through crowdfunding platforms. As the ‘silver consumers’, they can use sharing platforms to gain access to multitude of products or services. It can also help them safely navigate through the COVID-19 pandemic. One of the most recent publications, based on an in-depth study of the literature, states that [95]: “(…) the pandemic has caused new consumer segments such as the older generations to embark on online commerce, making this a potentially important issue.

Sharing platforms can be viewed as both a challenge and an opportunity. In the long run, their existence may help curb the human desire to possess things and the scale of consumption and ultimately change the lifestyles of all generations. It seems, therefore, that efforts be made to promote the advantages of sharing platforms among both young and mature consumers.

## Supporting information

S1 Questionnaire (DOCX)

S1 Dataset (XLSX)
