# Supplementary material for: Demographic and generational determinants of Poles’ participation in the sharing economy: Findings from a survey data analysis
Source: PLoS One. 2022 Jun 9;17(6):e0265341. doi: 10.1371/journal.pone.0265341 (PMC9182315; doi:10.1371/journal.pone.0265341)
Supplement: S1 Questionnaire — (DOCX) [file pone.0265341.s001.docx]

**Participation in the digital and sharing economy - questionnaire survey**

We are carrying survey about participation in the digital and sharing economy.

If the respondent hesitated, the interviewer explained and gave an example of the service (presented below in brackets).

1. Do you use electronic banking services via the Internet?

- Yes
- No

2. Do you use purchase / sale of goods via the Internet (e.g. allegro, OLX)?

- Yes
- No

3. Do you use free access to goods/services/knowledge/skills via the Internet (e.g. thingo)?

- Yes
- No

4. Do you use crowdfunding via the Internet (e.g. wspieramto)?

- Yes
- No

5. Do you use car sharing via the Internet (e.g. Uber, BlaBlaCar)?

- Yes
- No

6. Do you use accommodation booking via the Internet (e.g. CouchSurfing, Airbnb)?

- Yes
- No

7. Do you use outdoor equipment sharing and exchange via the Internet?

- Yes
- No

8. Do you use tours guided by locals via the Internet (e.g. SpottedbyLocals, Trip4real)?

- Yes
- No

**General Information**

1. Gender

- Men
- Women

2. Age group

- 18–24
- 25–29
- 30–34
- 35–39
- 40–44
- 45–49
- 50–54
- 55–59
- 60–64
- Over 65

3. Place of residence by voivodship

- Greater Poland
- Kuyavian-Pomeranian
- Lodz
- Lower Silesia
- Lubelskie
- Lubuskie
- Malopolskie
- Masovian
- Opolskie
- Podlaskie
- Pomerania
- Silesia
- Subcarpathian
- Świętokrzyskie
- Warmian-Masurian
- West Pomerania

Thank them for taking the time to answer the questionnaire.
